# Study of the Weathering Process of Gasoline by eNose

**DOI:** 10.3390/s18010139

**Published:** 2018-01-05

**Authors:** María José Aliaño-González, Marta Ferreiro-González, Gerardo F. Barbero, Jesús Ayuso, Miguel Palma, Carmelo G. Barroso

**Affiliations:** 1 Department of Analytical Chemistry, Faculty of Sciences, IVAGRO, University of Cadiz, Puerto Real, Cadiz P.O. Box 40 11510, Spain; mariajose.alianogonzalez@alum.uca.es (M.J.A.-G.); gerardo.fernandez@uca.es (G.F.B.); miguel.palma@uca.es (M.P.); carmelo.garcia@uca.es (C.G.B.); 2 Department of Physical Chemistry, Faculty of Sciences, University of Cadiz, Puerto Real, Cadiz P.O. Box 40 11510, Spain; jesus.ayuso@uca.es

**Keywords:** arson, characterization, chemometrics, eNose, evaporation, fingerprints, fire investigation, gasoline, ignitable liquids, weathering

## Abstract

In a fire investigation the rapid detection of the presence of ignitable liquids like gasoline is of great importance as it allows appropriate treatment of the remains, the identification of prevention methods and detects the possible presence of an arsonist. In some cases, analysts cannot access the fire scene in the first few hours due to the dangers involved in the situation and, as a consequence, phenomena such as weathering start. Ignitable liquid weathering is an evaporation process that results in an increase in the abundance of non-volatile compounds relative to volatile compounds, and this process changes the chemical composition. In the present work, the weathering of samples of gasoline at different times (from 0 h to a month) has been studied using an electronic nose (eNose). The influence of the volume used (40 µL and 80 µL) and the type of support (cork, wood, paper and cotton sheet) has been studied. Chemometric tools have been used with the aim of ascertaining the weathering time for which the developed method is capable of detecting the presence of gasoline. The eNose was able to discriminate samples of weathered gasoline. The support used for the samples did not seem to have an influence on the detection and the system.

## 1. Introduction

Fires (mainly forest fires) around the world and the consequent material and human damage are becoming a significant problem in our society, particularly given that human activity (accidentally or intentionally) is responsible for most cases. For this reason it is necessary to continue to study the capacity of new analytical tools and methodologies that can help investigators to determine the origin and cause of a fire, which is the ultimate goal of any fire investigation [1]. 

The most common ignitable liquids used as accelerants in arson attacks are gasoline, diesel and kerosene; especially the former due to it is ready availability and its more rapid combustion when compared to ignitable liquids [2,3,4].

After a fire is extinguished, analysis of the evidence remaining at the scene is a crucial step that can provide very valuable information on how the fire occurred. For instance, the detection of unexplained ignitable liquids, for example neat ignitable liquid (IL) adsorbed to different surfaces or recovered from the fire debris, can be crucial in determining whether the fire was deliberately started or not, that is, whether an act of arson is suspected [5,6].

Fire debris analysis is usually complicated due to the destructive nature of the fire, the high temperatures reached at the scene and the firefighting efforts. In addition, fire debris is usually subjected to different additional degradation processes such as evaporation or weathering, pyrolysis or microbial degradation. All of these phenomena can modify the chemical composition of the liquid and therefore its analysis, thus making it more difficult to identify the IL. In order to carry out a reliable interpretation of the results, the behavior of the different liquids under these processes must be studied. In particular, weathering is a degradation phenomenon in which the most volatile compounds of a mixture evaporate more quickly than non-volatile compounds, thus modifying the composition on the solid support [7,8]. In this way, the remaining amount of volatile compounds may be at levels below the limits of detection of the analysis methods while the compounds of lower volatility will be found at a relatively higher level. Weathering is closely related to variables, such as temperature, pressure, light and convection, and several studies have illustrated how these conditions affect the relative distribution of chemical residues [9,10,11]. This distribution has been used to estimate the age of ignitable liquids [12,13].

When an IL is used to start a fire, traces of the liquid may remain at the scene adhered to different materials [14]. These remaining liquid residues are not sampled until the fire has been extinguished and the security and toxic conditions at the fire scene are made safe. Depending on the magnitude of the fire, investigators may have to wait for minutes, hours or even days before accessing the scene, and during this time ILs can be suffering a weathering process that affects the fingerprint obtained. In addition, the different natures of the surfaces affected as the fire propagates and the volume of the accelerant used have a significant influence on the speed of the fire, the temperature reached and even in the method used to extinguish the fire [15,16]. For this reason, the influence of both the nature of the substrate and the IL volume in the weathering process must be studied. 

A large number of research and review articles have covered the different methods used for the determination and discrimination of ignitable liquids [4,17]. Different techniques have been used in the identification of ILs and these include Raman [18], thin layer chromatography (TLC) and high-performance thin layer chromatography (HPTLC) [19]. However, most common methods are based on gas chromatography (GC) [20,21,22,23] coupled with a flame ionization detector (FID) or, more recently, mainly mass spectra (MS) detectors [24]. These methods have been used for the identification of ILR (ignitable liquid residues) with good results, even in weathering conditions [4]. Indeed, the American Society for Testing and Materials (ASTM) standard E1618-11 provides guidelines for the identification and classification of ILRs from fire debris samples by gas chromatography–mass spectrometry (GC-MS) [17]. The identification of the ILS with GC-MS techniques is carried out by employing a data treatment that includes matching performance by visual inspection of the total ion chromatogram (TIC) and extracted ion chromatograms [25] combined with target compound analysis [26,27,28]. This technique provides good results but does suffer from some drawbacks [29] as it is based on the analyst’s experience and does not allow automation. 

Recently, the total ion spectrum (TIS) combined with chemometric methods has become a good alternative to TIC for identification and classification purposes [28,30,31,32]. TIS is equivalent to the average mass spectrum across the chromatographic profile and is independent of time, which means that it has advantages for inter-laboratory comparisons. In addition, this technique does allow automation. 

The development of non-separative techniques such as the electronic nose (eNose) has recently appeared as a promising alternative to chromatographic techniques, since in some cases it is sufficient to obtain a signal profile or fingerprint of the sample formed by all the components for identification purposes [33]. There are many types of eNose systems that have been applied with different purposes [34,35,36]. The main differences among them usually lie in the array of sensors used by each type. In this study, an alternative to the conventional sensor-based electronic noses, in particular an eNose based on mass spectrometry, was used. This eNose uses a quadrupole mass spectrometer as the detection system, so each mass-to-charge ratio (*m*/*z*) acts as a “sensor” and its abundance is equivalent to the sensor signal. The resulting mass spectra (MS) can be mathematically treated as an equivalent to the TIS and it is characteristic of each sample. As a consequence, suitable chemometric treatment of that signal can be easily developed as a fingerprint for each sample and used for rapid identification or characterization purposes. The eNose has been successfully optimized and validated for the analysis of ignitable liquids in fire debris and has provided characteristic fingerprints [37,38], for thermal desorption of ILR from carbon strips [1], for the discrimination of gasoline samples with different RON (Research Octane Number) [39,40], and for the characterization of neat ignitable liquids [41]. However, this technique has never been applied to the analysis of ignitable liquids after weathering.

The objective of the work described here was to study the evolution/modification of the eNose gasoline fingerprint after an evaporation weathering process for different times (from 0 h to 1 month) using different volumes and substrates of different natures in order to check the capacity of the eNose in the identification of weathered IL samples.

## 2. Materials and Methods

### 2.1. Samples

In order to assess the effect of the volume and the materials in a weathering process, a total of 72 samples were prepared by adding two different volumes of gasoline (40 µL and 80 µL) to four different substrates (pine wood, cork, paper and cotton sheet). The substrates used were square pieces of substrate with a width of 0.5 cm.

All samples were placed in 10 mL open vials (Agilent Crosslab, Santa Clara, CA, USA) inside the laboratory hood with accurate control of temperature (25 °C) and the flow of the laboratory hood was zero with the purpose of simulating natural conditions for the evaporation/weathering process.

Each support was placed in the middle of the base of the vial and the volume of gasoline was added to it. At different times (0 h, 6 h, 12 h, 24 h, 72 h, 1 week, 15 days, 21 days and a month) the vials with both volumes of gasoline for each type of substrate were closed and then analyzed. All samples were prepared in duplicate.

The samples were denoted as W when the support used was wood, C for cork, P for paper and S for cotton sheet. The volume and the time were also noted for each sample. For example, the first experiment with 40 µL in wood for 15 days was named “W_40 µL_15d_1”. The gasoline and the substrates used were purchased in local Spanish gas stations and stores, respectively. 

### 2.2. eNose Spectra Acquisition

Analysis of the weathered samples was performed on an Alpha Moss eNose system (Toulouse, France) composed of an HS 100 static headspace autosampler and a Kronos quadrupole mass spectrometer. Closed vials were placed in the autosampler oven to be heated at a temperature of 145 °C and agitated at 500 rpm in order to generate the headspace during 10 min. Headspace was extracted using a 5 mL gas syringe and injected into the mass spectrometer. The injection volume selected was 4.5 mL and the injection speed was 75 µL/s. The gas syringe was heated above the sample temperature (150 °C) to avoid condensation phenomena. Between each sample injection, the gas syringe was flushed with carrier gas (nitrogen) during 120 s at a fill speed of 100 µL/s to avoid cross-contamination. These conditions were previously optimized [35]. The total time per sample was approximately 12 min.

The components in the headspace of the vials were passed directly to the mass detector without any chromatographic separation or sample pre-treatment. In this way, for any given measurement, the resulting mass spectra (MS) give a fingerprint of the sample. Ion electron impact spectra were recorded in the range *m*/*z* 45–200. Instrument control was achieved using RGA (Residual Gas Analysis software package, Alpha Moss, Toulouse, France) and Alpha Soft 7.01 software (Alpha Moss, Toulouse, France).

### 2.3. Data Analysis

Mass spectra data were analyzed by hierarchical cluster analysis (HCA) and linear discriminant analysis (LDA). All these chemometric analyses were carried out using the statistical computer package SPSS 17.0 (SPSS Inc., Chicago, IL, USA).

## 3. Results and Discussion

### 3.1. Analysis of the Weathering Process

Firstly, a weathering study of gasoline supported on cotton sheet was carried out. These samples cover the two different volumes used for the gasoline (40 µL and 80 µL). Analyses of these samples were carried out over a period of four weeks. 

In order to remove noisy signals in the eNose system, any signal below 10% of the highest one at 0 h was not used in the study. The remaining signals were represented in mass spectra (MS), from which differences in the recorded signals during the study could be observed.

**Figure 1:**
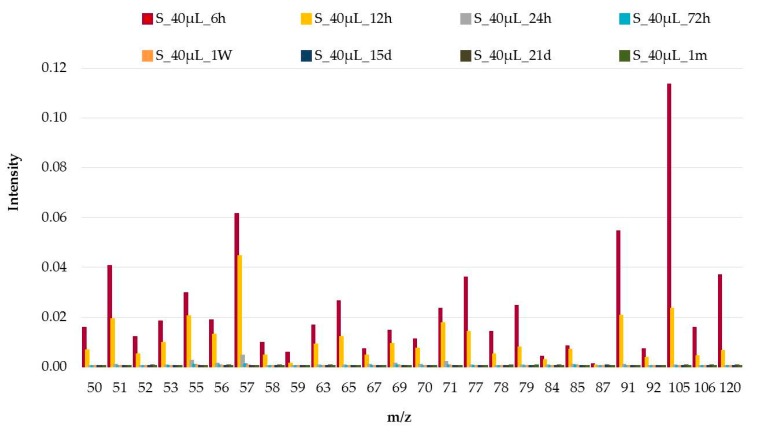
Mass spectra (MS) of samples of 40 µL of gasoline on cotton sheet (samples for 0 h are not included see Figure S1 in the Supplementary Materials for that result).

In the MS obtained on samples with 40 µL of gasoline, a modification in the intensities and their ratio can be observed according to the weathering time. This change was very significant between 0 and 6 h with a dramatic decrease in the intensities and the ratios of the signals (*m*/*z*) meaning that weathering process strongly starts during the first hours (Figure S1, Supplementary Materials). However, the same behavior can be seen in samples after 6 h of weathering. This finding is illustrated in Figure 1 for samples at 6 and 12 h where the ratio between the *m*/*z* (57/105) is below 1 in the case of 6 h but above 1 in the case of 12 h. Moreover, from 6 to 12 h, the intensity of *m*/*z* 57 decreases around 25%, while *m*/*z* 105 falls near 80%.

In the case of samples with 80 µL gasoline the gap between 0 and 6 h remains but the remaining intensities from samples after more than 6 h of weathering are higher than in the case of 40 µL. 

**Figure 2:**
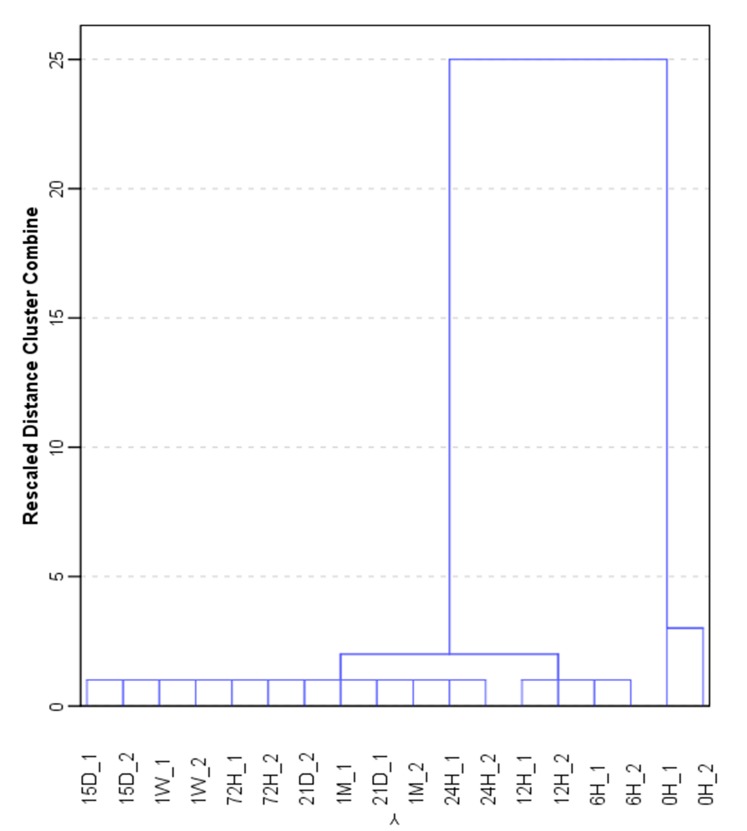
Dendrogram for 40 µL gasoline samples supported on cotton sheets for up to one month.

The evolution of the weathering process on gasoline samples was evaluated by carrying out a hierarchical cluster analysis (HCA). Signals (*m*/*z*) from samples with 40 µL of gasoline supported on cotton sheet as variable for forming groups. The method selected for this analysis was Ward’s method with a squared Euclidean distance. The results of the HCA are graphically represented in a dendrogram in Figure 2. As can be observed two main different groups were obtained: one at 0 h and the second for the rest of the samples, in which samples at 6 h and the samples at 12 h are distinguished from samples at 24 h and a month (Figure 2). In this case, the first group is useful to distinguish samples at 6 h and samples at 12 h, whereas the second group distinguishes the rest of the samples. It can be assumed that the weathering process starts very rapidly and dramatic changes occur after 6–12 h. 

A supervised technique, specifically a linear discriminant analysis (LDA) was run with the aim of studying the grouping properties of the samples. Based on the HCA results, two groups were selected. The first group covers samples from 6 h to 12 h of weathering (a total of four experiments) and the second group includes samples from 24 h to one month of weathering (a total of 12 experiments).

The results of the LDA show a classification of 100% confirming that two main groups can be found in the samples. The first group contains the samples at 6 and 12 h of weathering (four samples) while the second group covers samples after 24 h of weathering (12 samples). It was determined that after 24 h the signals from the remaining gasoline from the samples were so similar that differences could not be found. This means, a similar behavior for all signals from the samples was obtained after 24 h of weathering. Additionally, those signals differed from the resulting ones for less than 24 h of weathering.

With the aim of studying the weathering process in more detail, the *m*/*z* selected in the LDA as being significant were considered for each support and volume, and correlations between all of these *m*/*z* values were studied in order to identify the next *m*/*z* values for the development of intensity with time: 56, 57, 91, 106.

**Figure 3:**
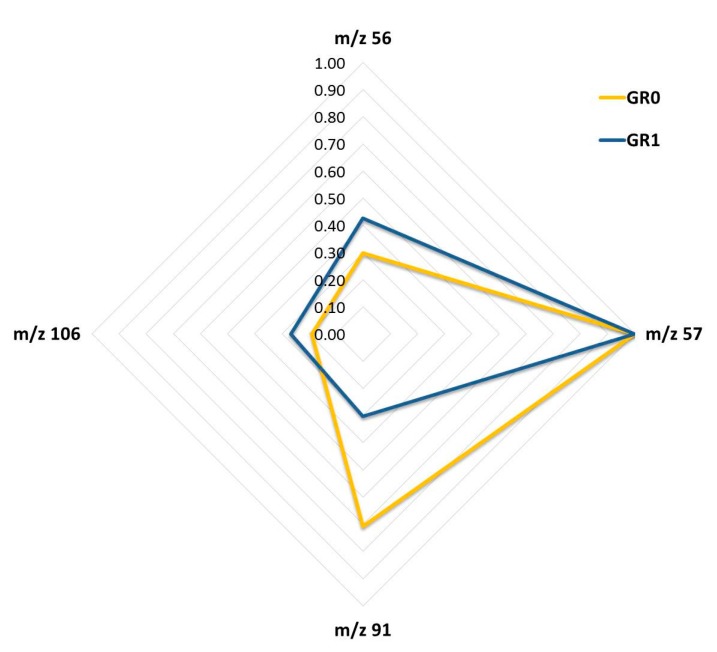
Spider Chart with the mass-to-charge ratio (*m*/*z*) values selected by the linear discriminant analysis (LDA).

The selected signals were used to generate a spider chart that allowed a fingerprint to be obtained for each kind of sample (Figure 3). The *m*/*z* values were normalized to the base peak at 100%. Two different fingerprints were obtained. It can be seen that in the first few hours (group 0) the signals at *m*/*z* 57 and 91 show the higher relative values and after 24 h, when the presence of a second pattern was detected, *m*/*z* 57 continued to show high levels, however *m*/*z* 56 and 106 became more significant. 

Based on these results, two different patterns could be identified in real samples—the first corresponding to the samples at the beginning of weathering and the second due to gasoline samples after an intense weathering process.

### 3.2. Influence of the Volume

Volume was considered as a potentially influential variable. In order to evaluate the effect on the signal when double the initial volume was used, similar experiments were carried out using 80 µL of gasoline on all of the supports. The results are shown in Table 1. 

The same procedures and analyses carried out on the 40 µL gasoline samples on cotton sheet were then carried out with 80 µL of gasoline. The initial intensity obtained on using a volume of 80 µL was markedly higher than that obtained on using 40 µL. Furthermore, the remaining signal was still similar to the initial signal after 24 h of weathering in the case of 80 µL samples. For the 40 µL, it must be noted that similar signals were obtained only up to 24 h, later moving to a different pattern. Therefore, the time taken to remove samples when attempting to detect gasoline on a supporting material is strongly dependent on the volume of gasoline in the material, because different signal patterns will be obtained depending on the time that elapses after gasoline was added to the solid material.

### 3.3. Influence of the Support

In order to study the influence of the surface of the support in conjunction with gasoline, three additional supports other than cotton sheet were selected (wood, cork and paper). 

A similar study than the previously described for cotton sheet, that is, first an HCA, later an LDA was applied for each supporting material. Finally, two different groups were obtained based on the specific resulting pattern obtained using the selected *m*/*z* from the LDA results. Table 1 shows the resulting homogenous groups defined for each support. The porosity of the material used did not appear to make any difference in the weathering process, that is, the nature of the supporting material for the sample was unimportant, as the eNose system had the same capability to discriminate between similar groups. In other words, the nature of the supporting material was not a key factor, and it was possible to determine gasoline residues in samples using two different patterns (Table 1). 

### 3.4. Study of the Kinectics

In order to analyze the influence that weathering time has on the final results from the analysis in the eNose system a kinetic study was carried out. In this respect, the *m*/*z* values highlighted as being important in the LDA were selected and normalized by assigning one unit to the *m*/*z* with the maximum intensity.

**Figure 4:**
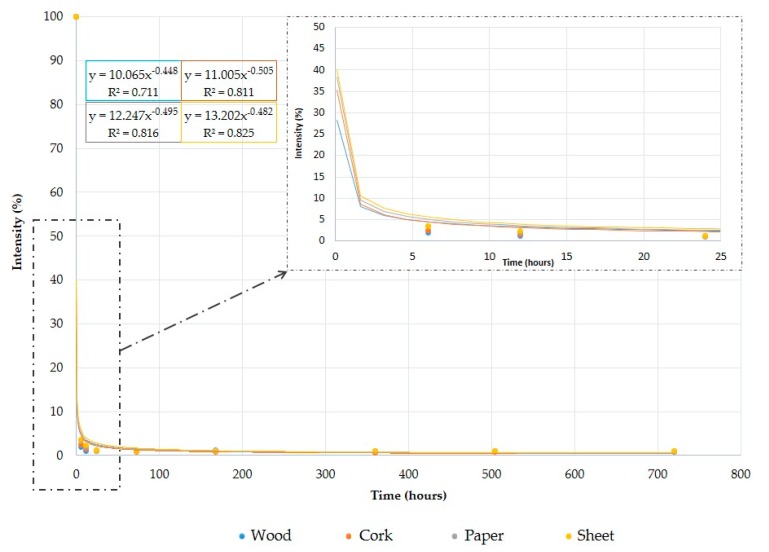
Analysis of the degradation rate for 80 µL of gasoline on different supports.

The effects of different volumes were analyzed on different supports. In all cases, the resulting signals for the selected *m*/*z* values were added and plotted against the analysis time (Figure 4). The rate of this kind of degradation did not adjust exactly with classical kinetics as either zero, first or second order due to the large differences in intensities between samples at 0 h and 6 h. However, it was found that the best mathematical adjustment for this kind of rate study was a potential adjustment, as a large gap was observed between 0 and 6 h followed by a progressive diminution to an almost constant value. 

The same procedure was applied to 40 µL and 80 µL samples, with the difference that after 24 h of weathering the remaining signal due to gasoline has a higher intensity in the case of 80 µL. Even after one month of weathering the eNose system was able to detect some signals similar to those detected after 24 h.

The effect of different supporting materials was not significant, that is, the resulting kinetics were very similar regardless of the supporting material. Thus, ultimately, the most interesting working variable to characterize the samples is the weathering time, because of different patterns must be used at different times.

## 4. Conclusions

The results obtained in this work showed that the eNose is a good analytical technique for the study of the gasoline weathering process.

The results indicate that the time of sampling of evidence is crucial in environments where a gasoline spill is suspected. It was observed that after 6 h the gasoline had lost most of its signal intensity and after this time it changed dramatically. Therefore, depending on the sampling time, a different pattern must be considered to identify gasoline in the solid support.

Small differences in the amount of gasoline used (40 or 80 µL) lead to a slow process of differentiation of signals from the initial case, and this requires longer analysis times to identify the presence of gasoline. 

Finally, the eNose system was able to discriminate samples of weathered gasoline regardless of the support used.

## Figures and Tables

**Table 1:** Groups of samples providing a 100% correct classification in the LDA for different sample volumes and different supporting materials.

Support	Volume Used/µL	Groups
Wood	40	Group 0: 6–24 h
		Group 1: 72 h–1 month
	80	Group 0: 6–24 h
		Group 1: 72 h–1 month
Cork	40	Group 0: 6–12 h
		Group 1: 24 h–1 month
	80	Group 0: 6–24 h
		Group 1: 72 h–1 month
Paper	40	Group 0: 6–12 h
		Group 1: 24 h–1 month
	80	Group 0: 6–24 h
		Group 1: 72 h–1 month
Sheet	40	Group 0: 6–12 h
		Group 1: 24 h–1 month
	80	Group 0: 6–24 h
		Group 1: 72 h–1 month

## Supplementary Materials

The following are available online at http://www.mdpi.com/1424-8220/18/1/139/s1, Figure S1: MS of 0 h and 6 h samples of 40 μL of gasoline on cotton sheet.
