# Supplementary material for: Study of the Weathering Process of Gasoline by eNose
Source: Sensors (Basel). 2018 Jan 5;18(1):139. doi: 10.3390/s18010139 (PMC5795821; doi:10.3390/s18010139)
Supplement: Supplementary file 1 [file sensors-18-00139-s001.pdf]

Study of the Weathering Process of Gasoline by eNose

María José Aliaño-González, Marta Ferreiro-González, Gerardo F. Barbero, Jesús Ayuso, Miguel Palma and Carmelo G. Barroso

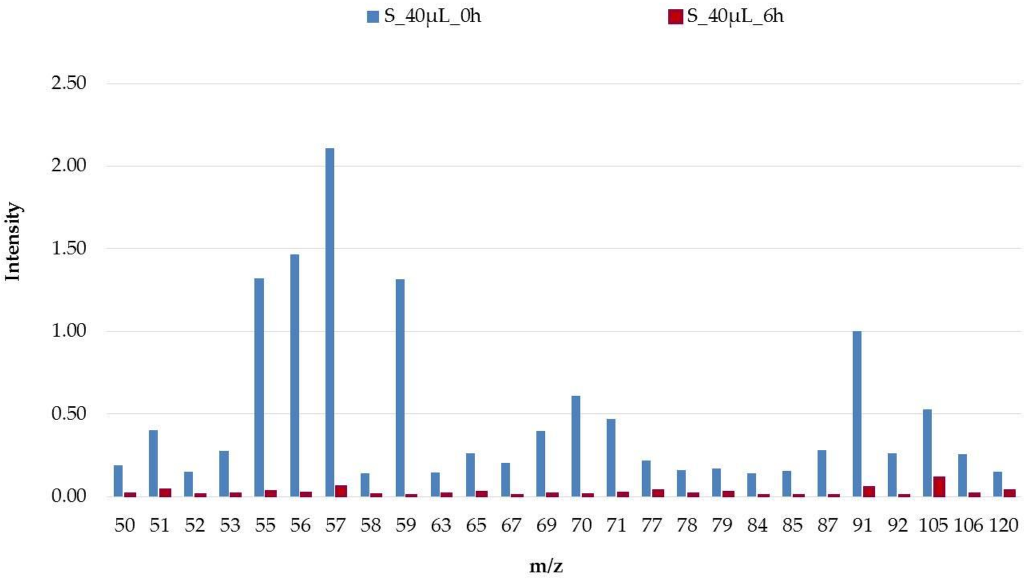

Figure S1. MS of 0 h and 6 h samples of 40 µL of gasoline on cotton sheet.
